# Editorial for the *IJMS* Special Issue “Progress in Understanding of Cardiac Arrhythmia Mechanisms and Antiarrhythmic Targets”

**DOI:** 10.3390/ijms24119134

**Published:** 2023-05-23

**Authors:** Narcis Tribulova

**Affiliations:** Center of Experimental Medicine, Slovak Academy of Sciences, Institute for Heart Research, 841 04 Bratislava, Slovakia; narcisa.tribulova@savba.sk

Cardiac rhythm disorders, in particular life-threatening ventricular fibrillation and stroke-provoking fibrillation of the atria, are a permanent focus of both clinical and experimental cardiologists. This is because of their high impact on mortality and morbidity worldwide, despite the progress of both research and treatment in recent decades.

Atrial fibrillation is most frequent arrhythmia, whose incidence increases in older populations, while affecting both men and women. Ventricular fibrillation can be a cause of sudden cardiac death sometimes, even in patients with an implantable cardioverter–defibrillator or often in athletes.

Both invasive and non-invasive approaches are still not efficient enough to prevent the occurrence and/or recurrence of these cardiac arrhythmias. Therefore, it is challenging to address this topic at both the bench and bedside molecular levels to understand cardiac arrhythmogenesis comprehensively. This is condition sine qua non to discover novel targets and approaches aimed at preventing and/or attenuating development of cardiac arrhythmias in various pathophysiological conditions, namely those accompanied by oxidative stress and chronic systemic inflammation, such as hypertension, obesity, diabetes mellitus or dislipidemia. In this context, it is important to emphasize that lifestyle modifications may have a great impact on cardio-protection and may significantly attenuate the vulnerability of the heart to arrhythmias.

This Special Issue “Progress in Understanding of Cardiac Arrhythmia Mechanisms and Antiarrhythmic Targets” covers a selection of recent research topics and updated review articles based on the molecular and cellular view of cardiac arrhythmias and their possible prevention. Papers published in this Special Issue provide relevant pieces of the mosaic aiming to preserve the synchronized electro-mechanical function of heart muscle.

The main messages from the papers highlight:

Pharmacological approaches targeting multiple mechanisms besides oxidative stress might be more effective in the treatment of ventricular arrhythmias than current antiarrhythmic therapy [1].

The pathogenic impact of very low-density lipoprotein on atrial remodeling and vulnerability to atrial fibrillation [2].

QRS complex patterns in left ventricular hypertrophy could be potentially recognized for predicting ventricular arrhythmia and for monitoring the effect of therapy [3].

Thyroid hormones should be considered a biomarker for cardiac arrhythmia screening and their tailored management because of their multifaceted cellular action [4]. 

The sympathetic nervous system should be a target in the management of sudden cardiac death in patients with heart failure [5]. 

Micro-ribonucleic acids, short non-coding RNA molecules, responsible for the regulation of gene expression seem to be potential screening biomarkers for atrial fibrillation and even treatment targets for this arrhythmia [6].

The delayed antiarrhythmic effect of sodium nitrite can be explained by the attenuation of calcium overload, directly by modulating ion channels or indirectly by reducing the mitochondrial reactive oxygen species production [7].

Adenosine receptors and activation protein kinase C are involved in the mechanisms of ischemic post-conditioning-induced protection against ventricular fibrillation in isolated heart preparation [8].

The melatonin-related antiarrhythmic effect is associated with its oxidative stress-independent action on ventricular activation [9].

The arrhythmogenic potential of β1-adrenoceptor autoantibodies and their susceptibility to ventricular fibrillation are attenuated by the supplementation of hypertensive rats with omega-3 fatty acids, which is associated with the up-regulation of connexin-43 [10].

Human variants of plakophilin-2 are associated with familial arrhythmogenic cardiomyopathy, while exercise upon plakophilin-2 deficiency induces pro-arrhythmic cardiac remodeling, likely based on impaired Ca^2+^ cycling and electrical conduction [11].
